# Retrotransposon vectors for gene delivery in plants

**DOI:** 10.1186/1759-8753-1-19

**Published:** 2010-08-02

**Authors:** Yi Hou, Jyothi Rajagopal, Phillip A Irwin, Daniel F Voytas

**Affiliations:** 1 Department of Genetics, Cell Biology and Development and Center for Genome Engineering, University of Minnesota, Minneapolis, Minnesota USA 55455, USA; 2 Department of Statistics, University of Texas, San Antonio, San, Antonio, TX 78249, USA; 3 Complete Genomics, Inc., 2071 Stierlin Court, Mountain View, CA 94043, USA; 4 VWR International, LLC, 1310 Goshen Parkway, West Chester, PA 19380, USA

## Abstract

**Background:**

Retrotransposons are abundant components of plant genomes, and although some plant retrotransposons have been used as insertional mutagens, these mobile genetic elements have not been widely exploited for plant genome manipulation. In vertebrates and yeast, retrotransposons and retroviruses are routinely altered to carry additional genes that are copied into complementary (c)DNA through reverse transcription. Integration of cDNA results in gene delivery; recombination of cDNA with homologous chromosomal sequences can create targeted gene modifications. Plant retrotransposon-based vectors, therefore, may provide new opportunities for plant genome engineering.

**Results:**

A retrotransposon vector system was developed for gene delivery in plants based on the Tnt1 element from *Nicotiana tabacum*. Mini-Tnt1 transfer vectors were constructed that lack coding sequences yet retain the 5' and 3' long terminal repeats (LTRs) and adjacent *cis *sequences required for reverse transcription. The internal coding region of Tnt1 was replaced with a neomycin phosphotransferase gene to monitor replication by reverse transcription. Two different mini-Tnt1 s were developed: one with the native 5' LTR and the other with a chimeric 5' LTR that had the first 233 bp replaced by the CaMV 35 S promoter. After transfer into tobacco protoplasts, both vectors undergo retrotransposition using GAG and POL proteins provided in *trans *by endogenous Tnt1 elements. The transposition frequencies of mini-Tnt1 vectors are comparable with native Tnt1 elements, and like the native elements, insertion sites are within or near coding sequences. In this paper, we provide evidence that template switching occurs during mini-Tnt1 reverse transcription, indicating that multiple copies of Tnt1 mRNA are packaged into virus-like particles.

**Conclusions:**

Our data demonstrate that mini-Tnt1 vectors can replicate efficiently in tobacco cells using GAG and POL proteins provided in *trans *by native Tnt1 elements. This suggests that helper Tnt1 constructs can be developed to enable a Tnt1-based two-component vector system that could be used in other plant species. Such a vector system may prove useful for gene delivery or the production of cDNA that can serve as a donor molecule for gene modification through homologous recombination.

## Background

Long terminal repeat (LTR) retrotransposons are mobile genetic elements that replicate through reverse transcription of a messenger (m)RNA intermediate. Because LTR retrotransposons and retroviruses are similar in their genetic organization and mechanism of replication [1], they are collectively referred to as retroelements. The LTRs delimit retroelement insertions, and contain the promoters and transcription terminators required to produce a retroelement mRNA. Adjacent to the 5' and 3' LTRs are a primer binding site (PBS) and a polypurine tract (PPT), respectively, which serve as priming sites for reverse transcription. All retroelements have two genes in common: *GAG *encodes proteins that form virus or virus-like particles (VLPs) and *POL *encodes three enzymes, namely reverse transcriptase (RT), integrase (IN) and protease (PR).

Retroelement replication begins with the synthesis of an element mRNA that is translated into protein and also serves as the template for reverse transcription into cDNA. After translation, GAG forms virus or VLPs into which are packaged retroelement mRNAs and the *POL *gene products. Reverse transcription by RT is initiated at the PBS using a host-encoded transfer (t)RNA or a tRNA-derived primer. Minus-strand DNA synthesis extends from the PBS to the 5' end of the mRNA to generate minus-strand strong stop DNA. Because of sequence redundancy between the 5' and 3' LTRs, the strong stop DNA jumps to the 3' end of the same or a different mRNA by pairing with complementary sequences. DNA synthesis then proceeds to the 5' end of the mRNA, completing the minus-strand synthesis. The RNaseH activity of RT removes RNA from the RNA-DNA hybrid, with the exception of RNA paired to the PPT, which serves as the plus-strand primer. When the plus-strand DNA reaches the end of the template, it undergoes a second jump by pairing with sequences at the 5' end of the cDNA. Completion of plus-stand synthesis generates a full-length, double-stranded cDNA that is the substrate for integration. The cDNA is carried into the nucleus by the integration complex, the major component of which is integrase. Integrase cuts the genome and inserts the cDNA into the host DNA.

The ability to catalyze cDNA integration makes retroelements a good tool for gene delivery [2]. Retroelement-based gene delivery systems typically use a two-component strategy. One component is a transfer vector, which carries a foreign gene for delivery to the host genome. The transfer vector also has all the *cis*-regulatory sequences required for high-efficiency replication. The second component is a helper retroelement, which supplies the necessary proteins required for replication in *trans*. When a cell expresses both helper and vector elements, functional replication intermediates are formed. For the retroviruses, these intermediates are virus particles that can be harvested and used to infect cells or tissues to deliver the target gene. For the retrotransposons, the replication intermediates are VLPs that carry a copy of the vector retroelement mRNA. This mRNA is reverse transcribed into cDNA, which can then integrate or recombine with the host genome.

Two-component retroviral vector systems are widely used for gene delivery to human cells, because of their ability to maintain persistent long-term expression of the transgene [2]. Two-component retrotransposon gene delivery systems have also been developed in budding yeast [3]; however, retrotransposon-based two-component vectors have not been widely used for gene delivery outside of the yeast model.

LTR retrotransposons are particularly abundant in plant genomes [4], and the first transpositionally competent plant retrotransposon described was the tobacco Tnt1 element [5]. Mobility of Tnt1 is greatly increased by stresses such as protoplast isolation, cell culture and pathogen attack [6,7]. For example, newly transposed Tnt1 copies have been detected in nearly 25% of tobacco plants regenerated from protoplast culture [8]. It was also shown that Tnt1 elements prefer to integrate within or near gene coding sequences, and that these insertions are typically well-tolerated by the host [9]. Furthermore, Tnt1 elements have high transpositional competence in various heterologous plant species, including *Arabidopsis thaliana*, *Medicago truncatula *(barrel clover) and *Lactuca sativa *(lettuce) [10-12].

In this report, we describe a Tnt1-based vector system that is designed to replicate and deliver foreign genes of interest to plants. Tnt1 transfer vectors (mini-Tnt1 elements) were constructed by replacing most of the Tnt1 coding sequence with the selectable marker neomycin phosphotransferase (NPT)II. Tnt1-encoded gene products required for transposition were provided in *trans *by endogenous Tnt1 elements, whose expression was induced by protoplast isolation. We show that mini-Tnt1 elements lacking functional gene products can be effectively complemented in *trans *by the endogenous helper Tnt1 elements. Our results suggest that, like the vertebrate retroviruses, LTR retrotransposons can be engineered to be efficient vehicles for gene delivery in plants.

## Results

### A mini-Tnt1 transfer vector

**Figure 1:**
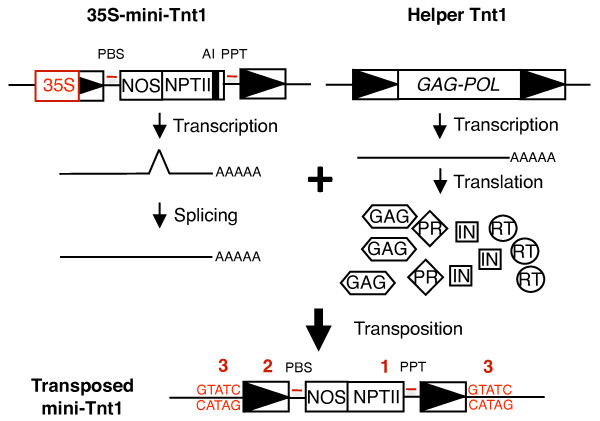
**The mini-Tnt1 vector system**. Mini-Tnt1 is a non-autonomous element that carries a neomycin phosphotransferase gene (NPTII) driven by the nopaline synthase (NOS) promoter. An artificial intron (AI; black box) is present in the NPTII coding sequence. Long terminal repeat (LTR) sequences are shown as open boxes with solid triangles. The 35S-mini-Tnt1 has a cauliflower mosaic virus 35 S promoter that replaces part of the 5' LTR (red). The primer binding site (PBS) and polypurine tract (PPT) are depicted by red bars. Below the 35S-mini-Tnt1 is the element mRNA that undergoes splicing and serves as a template for reverse transcription. To the right of 35S-mini-Tnt1 is an autonomous Tnt1 retrotransposon present in the tobacco genome (not drawn to scale). The autonomous Tnt1 provides GAG and POL in *trans *to enable transposition of the mini-Tnt1 mRNA. *POL *gene products include protease (PR), reverse transcriptase (RT) and integrase (IN). The replicated mini-Tnt1 at the bottom of the figure has several features indicative of transposition (red numbers): (1) it lacks an intron,;( 2) it has a complete 5' LTR that is reconstituted during reverse transcription, and (3) it is flanked by a target site duplication (red DNA sequences).

Tnt1 was amplified by PCR from tobacco genomic DNA and modified by oligonucleotide-directed mutagenesis so that it was identical to the published Tnt1 sequence (GenBank accession number X13777). A mini-Tnt1 vector was constructed for DNA delivery, with complete 5' and 3' LTRs, which provide transcription initiation and termination sites, respectively (Figure 1). Sequences within the 5' internal domain of Tnt1, which extend into the first 315 bp of *GAG*, were included. Non-coding internal domain sequences near the 3' LTR were also included. The internal domain sequences should contain the *cis*-acting elements (for example, priming sites) required for reverse transcription [3,13]. A modified version of the mini-vector was generated in which the 5' region of the 5' LTR (U3) was replaced with the cauliflower mosaic virus (CaMV) 35 S promoter (hereafter referred to as 35S-mini-Tnt1). The CaMV 35 S promoter should enable constitutive mini-Tnt1 expression [14].

An *NPTII *gene with a nopaline synthase (NOS) promoter was cloned into both mini-Tnt1 vectors. Plant cells expressing NPTII are kanamycin-resistant (Kan^r^). The *NPTII *gene also contains an 85 bp artificial intron (AI), which is in the same orientation as the mini-Tnt1 transcript. When mini-Tnt1 vectors are transferred into tobacco protoplasts, the endogenous Tnt1 elements, whose expression is induced by protoplast isolation [15], should provide the gene products required for mini-Tnt1 retrotransposition in *trans*. Versions of mini-Tnt1 and 35S-mini-Tnt1 with deletions of the PBS were constructed to serve as negative controls. Because transposition of yeast retrotransposon Ty1 was abolished in similar PBS mutants [16], it was expected that these mutants would disrupt the mini-Tnt1 transposition.

The mini-Tnt1 constructs provide three ways to monitor reverse transcription and retrotransposition (Figure 1). First, after splicing of the mRNA and reverse transcription, the intron of NPTII should not be present in the newly transposed elements. This enables parental and progeny elements to be distinguished by PCR. Secondly, during transposition, a complete 5' LTR of the 35S-mini-Tnt1 element will be regenerated, because U5 from the 3' LTR in the mRNA is used as a template for the synthesis of U5 in the 5' LTR of the newly transposed element. Reconstitution of the 5' LTR in a newly transposed 35S-Tnt1 element in *Arabidopsis *has previously been demonstrated [11]. Third, if mini-Tnt1 elements undergo transposition, the flanking sequences of new insertions should contain a characteristic 5 bp duplication of the integration site [5].

### Mini-Tnt1 cDNA synthesis

**Figure 2:**
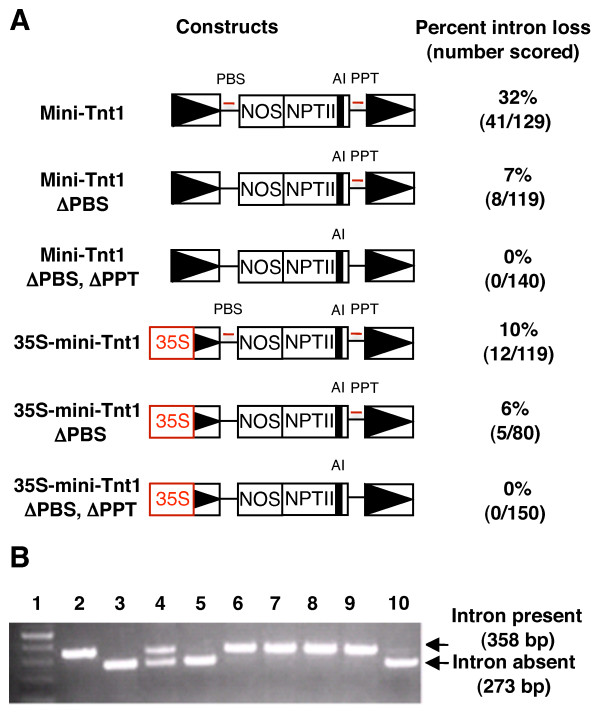
**cDNA synthesis by mini-Tnt1**. **(A) **Schematics of mini-Tnt1 constructs that were introduced into tobacco protoplasts. LTR sequences are shown as boxes with solid triangles. The thin red lines denote locations of the PBS and PPT. The artificial intron (AI) is depicted by a small black box. Kanamycin-resistant (Kan^r^) calli were obtained for each construct and scored by PCR for the loss of the intron in NPTII. Percentage intron loss is the number of Kan^r ^calli with complementary (c)DNA (as measured by the absence of the intron) divided by the total number of calli scored, multiplied by 100. Exact numbers of events scored are indicated in parentheses below the percentages. **(B) **Typical results of the PCR screen for intron loss among Kan^r ^calli. Lane 1 = molecular length markers; lane 2 = PCR results using a control template that has the intron; lane 3 = PCR results using a control template without an intron; lanes 4-10 = PCR results from DNA samples derived from individual Kan^r ^calli.

Tobacco protoplasts were electroporated separately with the mini-Tnt1 and 35S-mini-Tnt1 constructs, and with negative control constructs with the PBS deletions (Figure 2A). Each transformation contained approximately 2 × 10^6 ^protoplasts. Endogenous Tnt1 elements are expressed during protoplast isolation [15], and therefore should provide GAG and POL proteins, which are required for the replication of the mini-Tnt1 elements. After electroporation, the protoplasts were allowed to grow for 2 months, then Kan^r ^calli were selected and scored. Kanamycin resistance can arise either by integration of the mini-Tnt1 constructs into the genome, or by expression, replication and integration of mini-Tnt1 cDNA. To distinguish between these possibilities, genomic DNA was isolated from Kan^r ^calli and amplified by PCR with primers flanking the intron. If mini-Tnt1 cDNA is synthesized, the loss of the intron will be detected because it will generate a PCR product 85 bp shorter than the fragment with the intron (Figure 2B). For mini-Tnt1, about one-third of the calli tested had a PCR product corresponding to the size expected due to splicing (Figure 2A; see Additional file 1). For the 35S-mini-Tnt1 construct, about one in 10 calli showed evidence of intron loss. Sequence analysis of the smaller PCR fragments indicated that the intron had indeed been spliced, thereby demonstrating that mini-Tnt1 cDNA is synthesized (data not shown).

Of the samples showing evidence of splicing, many had two PCR products (Figure 2B). The larger (unspliced) product probably derives from the parental mini-Tnt1 elements, which integrated into the plant genome by non-homologous end-joining or by homologous recombination with endogenous Tnt1 elements, whereas the smaller PCR product may correspond to progeny mini-Tnt1 elements that transposed into the genome after reverse transcription. Approximately half of the samples yielded only a single PCR product of the size predicted for splicing of the intron. This suggests that mini-Tnt1 cDNA synthesis and insertion into the genome occurred in the absence of insertion of the parental mini-Tnt1 construct.

To confirm that reverse transcription of mini-Tnt1 was responsible for the observed intron loss, PCR screens were performed with Kan^r ^calli obtained with the PBS mutants. We anticipated that the PBS mutations would compromise transposition because they would fail to prime first strand DNA synthesis; however, intron loss was still detected in both mutant mini-Tnt1 constructs (Figure 2A). During protoplast isolation, it is known that the transcription of endogenous Tnt1 elements is greatly increased [17]. Because multiple copies of the retrotransposon mRNA can be packaged into VLPs [18,19], it is possible that VLPs contain mRNAs derived from both endogenous Tnt1 and PBS mutants. If minus-strand strong stop DNA is generated from the endogenous Tnt1 mRNA, it could be transferred to the mRNA of a mini-Tnt1 element with the PBS mutation during the first strand transfer. Second strand cDNA synthesis would then be primed from the PPT on the mini-Tnt1 mRNA. To test whether template switching could explain our results, a double mutant mini-Tnt1 that lacks both the PBS and the PPT was created (Figure 2A). Kan^r ^calli derived from the double mutants were analyzed, and no evidence of intron loss was obtained. These experiments indicate that the observed intron loss is due to reverse transcription and support our hypothesis that more than one mRNA serves as a template in the synthesis of mini-Tnt1 cDNA.

### Mini-Tnt1 elements transpose in tobacco protoplasts

**Figure 3:**
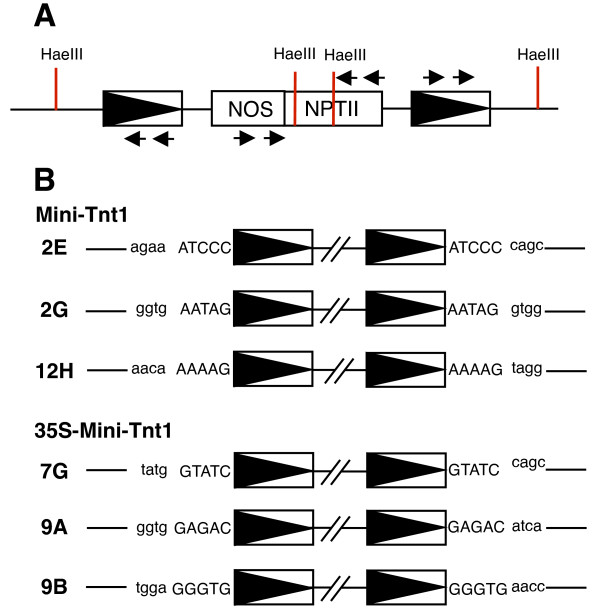
**Integration sites of mini-Tnt1s**. **(A) **Genomic DNA from calli with putative transposition events was digested with the restriction enzyme *Hae*III. After intramolecular ligation of the *Hae*III fragments, two pairs of primers (indicated by the horizontal arrows) specific for the 5' or 3' long terminal repeats (LTRs), the neomycin phosphotransferase gene (NPTII), or the nopaline synthase (NOS) promoter. were used in separate inverse PCR assays. **(B) **Sequences flanking six mini-Tnt1 insertions are shown in lowercase letters (three from mini-Tnt1 and three from 35S-mini-Tnt1), and duplicated sequences at the insertion site in uppercase letters.

We next sought to determine whether the PCR products without introns were derived from transposition events or from cDNA that had recombined into the genome. Sequences flanking mini-Tnt1 insertions were recovered by inverse (I)PCR [20] and examined for target site duplications characteristic of transposition. Ten Kan^r ^calli (four derived from the 35S-mini-Tnt1 construct and six derived from mini-Tnt1) were analyzed. In all 10 samples, there was no evidence that the parental Tnt1 plasmid had integrated into the tobacco genome (that is, PCR assays revealed that the intron in NPTII had been spliced). Genomic DNA from the calli was digested with *Hae*III, which has recognition sites in NPTII but not the mini-Tnt1 LTR. Digestion with *Hae*III, therefore, releases complete 5' and 3' LTRs and part of the sequence of NOS-NPTII (Figure 3A). Oligonucleotides complementary to the LTR and NOS-NPTII were then used as primers for IPCR. Selective amplification of the mini-Tnt1 element (and not endogenous elements) was ensured by using NOS-NPTII primers. Nested PCR reactions were preformed to increase the fidelity of the reaction. PCR products were cloned and sequenced, and in six of the samples, insertions were identified that were flanked on both sides by 5 bp target site duplications (Figure 3B). Additional target site sequences were identified in these six samples, suggesting that the mini-Tnt1 transposed more than once (data not shown). For the remaining four samples, flanking sequences for only a single 5' or 3' LTR were recovered, perhaps due to the inability of the PCR to adequately amplify one side of the insertion because the nearest *Hae*III site in the genomic DNA was a considerable distance from the insertion site. We conclude that mini-Tnt1 elements, like their full-length progenitors [5], create a duplication of 5 bp upon integration into the tobacco genome.

The tobacco genome harbors several hundred copies of Tnt1 [9], and it is possible that the integrated mini-Tnt1 elements described above resulted from homologous recombination between mini-Tnt1 cDNA and endogenous Tnt1 elements. If homologous recombination occurred, then the target site duplications described above would have arisen from pre-existing Tnt1 insertions. We characterized the target sites in wild-type tobacco genomic DNA by performing PCR with primers located upstream and downstream of mini-Tnt1 insertion sites. The wild type insertion sites were rescued, and all six showed no evidence of a pre-existing Tnt1 (data not shown), consistent with the mini-Tnt1 s having integrated (not recombined) into the tobacco genome. The sequence of each mini-Tnt1 target site was searched against the DNA sequence databases. Sequence similarity with a cut-off e-value of < 1.00E-5 was considered significant. Four of the six insertions showed similarity to previously characterized DNA sequences (Table 1). Three were within coding sequences, and one was in a retrotransposon from the related species *Solanum demissum*. The apparent preference for insertion of mini-Tnt1 s into coding sequences is similar to that described for the native Tnt1 retrotransposon [9].

**Table 1:** BLAST search results for sequences at mini-Tnt1 insertion sites.

Parental element(insertion)	Sequence with most similarity to insertion site	Score(e-value)	Location of insertion
Mini-Tnt1(2E)	AM792845: Glutathione-regulated potassium-efflux system protein from *Verminephrobacter eiseniae*	4.2^e-19^	Within coding sequence

Mini-Tnt1(2G)	None identified	NA	

Mini-Tnt1(12H)	AY3139395: 5-epi-aristolochene synthase in *Nicotiana tabacum*	4.8^e-67^	Within coding sequence

35S-mini-Tnt1(7G)	BP131841: Homolog to sensor protein in *Bacillus *sp.	5.4^e-22^	Within coding sequence

35S-mini-Tnt1(9A)	None identified	NA	

35S-mini-Tnt1(9B)	AM811601: GAG-POL protein in *Solanum demissum*	2.0^e-10^	Within coding sequence

NA = not applicable.

**Figure 4:**
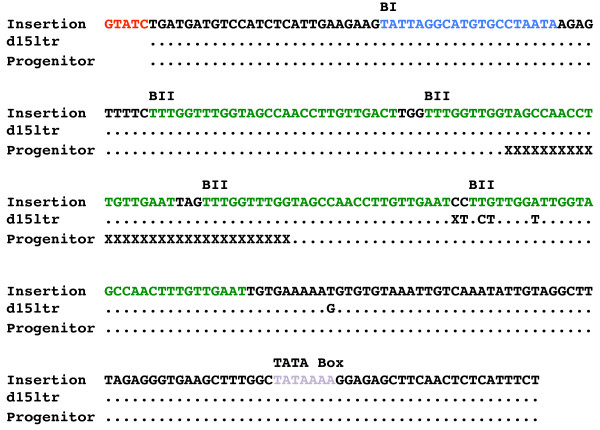
**Evidence for reconstitution of the 35S-mini-Tnt1 5' long terminal repeat (LTR), after transposition and for template switching between mini-Tnt1 and endogenous Tnt1 mRNA**. **(A) **The alignment depicts the DNA sequence for the U3 region of the 5' LTR for three different Tnt1 elements. The top row is the sequence of a newly transposed, mini-35S-Tnt1 element. Full stops (periods) = identical residues; X = missing sequences; blue sequences = BI repeats; green sequences = BII repeats; red sequences = target site duplication; purple sequences = TATA box. The middle sequence is from the previously described d15ltr insertion [8], which shares sequence similarity to the newly transposed mini-Tnt1. The bottom sequence is from the progenitor mini-Tnt1. Note the absence of the 35 S promoter in the newly transposed 35S-mini-Tnt1. Note also the presence of an extra BII repeats in the newly transposed mini-Tnt1, which was probably acquired by template switching with mRNA from a native Tnt1 element.

The mRNA template used by LTR retrotransposons for reverse transcription begins at the junction between the U3 and R regions in the 5' LTR, and ends at the junction between R and U5 in the 3' LTR. If mini-Tnt1 replicates using the same mechanism as retroviruses and other retrotransposons, then we expected newly integrated 35S-mini-Tnt1 elements to lack the 35 S promoter and to have complete 5' LTRs with U3, R and U5 (Figure 1). We investigated reconstitution of the 5' LTR in four 35S-mini-Tnt1 insertions that were recovered by IPCR. DNA sequencing revealed that in all four cases, the 5' LTRs were reconstituted through reverse transcription (data not shown and Additional file 1). For three of these, the sequence was consistent with the use of the mini-Tnt1 U5 region in the 3' LTR as a template; however, the remaining 5' LTR was chimeric, and its sequence matched both the U3 region of the mini-Tnt1 and an endogenous Tnt1 variant with an extra BII repeat in U3 (Figure 4) [8]. The 3' LTR of this element with the chimeric 5' LTR was rescued by PCR using primers located downstream of the 3' LTR and within the 3' end of NPTII. DNA sequencing revealed that the 3' LTR was identical to the 5' LTR: a chimera between the U3 region of the mini-Tnt1 5' LTR and an endogenous Tnt1 variant (see Additional file 1). It has long been known that reverse transcriptase can switch between templates during reverse transcription [21]. The chimeric LTR is consistent with the use of both mini-Tnt1 and endogenous element mRNA templates. The identification of chimeric elements derived from two different mRNA templates bolsters our previous predictions that Tnt1 can engage in template switching, as suggested by the transposition competence of the mini-Tnt1 elements with PBS mutations (Figure 2A).

## Discussion

### Mini-Tnt1 transposition in tobacco protoplasts

In this study, we demonstrated that two different mini-Tnt1 vectors are capable of transposition when introduced into tobacco protoplasts. Transcription of endogenous Tnt1 elements is activated by protoplasting, and *pol *gene products produced by endogenous Tnt1 elements can carry out cDNA synthesis and integration of mini-Tnt1 vectors. For the mini-Tnt1 vector with the complete 5' LTR, transposition events were detected in one-third of calli generated from protoplasts. Previous work demonstrated a 25% transposition frequency for endogenous Tnt1 elements when plants were regenerated from protoplasts in the absence of selection [8]. For the construct with the 35 S promoter (35S-mini-Tnt1), transposition was observed in about 10% of the transformed protoplasts. In 35S-mini-Tnt1, the U3 region of the 5' LTR was replaced with the 35 S promoter. U3 contains several *cis*-acting elements that behave as transcriptional activators [6]. In fact, the native 5' LTR was reported to have higher transcriptional activity than the 35 S promoter in a tobacco transient expression assay [15]. Because transcription is required for retrotransposition, higher transcription may explain why the mini-Tnt1 element with the native LTR transposed at a higher frequency than the 35S-mini-Tnt1.

Our results provide evidence that template switching occurs during Tnt1 reverse transcription. This indicates that multiple copies of mRNA are packaged into Tnt1 VLPs, consistent with previous work on both retroviruses and yeast LTR retrotransposons demonstrating that at least two molecules of mRNA are packaged into a single virus particle or VLP [18,19]. Two independent lines of experimental evidence suggested that template switching occurs during reverse transcription. First, cDNA was synthesized from the mini-Tnt1 elements with PBS deletions. We believe that cDNA synthesis occurs when minus-strand strong stop DNA derived from the endogenous Tnt1 mRNA transfers to the mini-Tnt1 mRNA with the PBS mutation. This is consistent with the observation that no cDNA was produced from the mini-Tnt1 constructs with both PBS and PPT mutations, because the PPT from the mini-Tnt1 would be required to complete reverse transcription. Secondly, one of the 5' LTRs of a newly transposed 35S-mini-Tnt1 had an extra BII repeat in U3, which was probably derived from U3 of the endogenous Tnt1. Additionally, U3 in the 3' LTR of the same transposed element had the identical sequence as the 5' LTR. This suggests that both endogenous Tnt1 and 35S-mini-Tnt1 mRNAs were packaged into one VLP, and that template switching occurred during minus strand DNA synthesis of 35S-mini-Tnt1. Recombination and template switching have been frequently observed in retroviruses [22]. High frequencies of deletion caused by recombination during reverse transcription were also observed during retrotransposition of Ty1 in *Saccharomyces cerevisiae *[23]. Recently, sequence analysis suggested that template switching occurred during reverse transcription of retrotransposons in the Triticeae tribe of the Pooideae subfamily, and this was evoked to explain how some complex LTR retrotransposon insertions arose [24]. Our evidence supports the emerging picture that multiple mRNAs are used during reverse transcription of diverse LTR retroelements, and that recombination and template switching are commonplace.

### Applications of a mini-Tnt1 two-component system

In the tobacco experimental system used in this study, the proteins required for mini-Tnt1 mobilization were provided by endogenous Tnt1 elements. In order for the mini-Tnt1 to be useful in other plant species, a helper Tnt1 element that expresses Tnt1 *GAG-POL *will be required. We performed preliminary experiments in which mini-Tnt1 vectors were introduced into *Arabidopsis *cells along with a construct that expresses Tnt1 *GAG-POL*. cDNA production was observed by PCR, using the intron-loss assay (see Additional file 1). The results suggest that the helper Tnt1 effectively substituted for the native Tnt1 elements in this heterologous system. We believe these preliminary experiments bode well for the generation of a Tnt1-based two-component system that can function in a variety of plant species.

Insertional mutagenesis is a powerful tool for forward genetic analysis in plants [25], and the most popular insertion elements are *Agrobacterium *T-DNA and class II transposable elements such as Ac, En/Spm and mutator of maize. T-DNA, however, is not particularly useful as an insertional mutagen in plants with large genomes, because it tends to integrate randomly; it also cannot be used in plants that are difficult to transform with *Agrobacterium*. Class II transposable elements move by a 'cut and paste' mechanism, and thereby often create unstable mutations. Tnt1 overcomes all these limitations because it prefers to integrate in or near coding sequences, and because it moves by a 'copy and paste' mechanism. Full-length Tnt1 elements have already been used successfully as insertional mutagens in *M. truncatula, Arabidopsis *and lettuce [10,11,26]. Mini-Tnt1 vectors should also be useful for gene tagging for several reasons: (1) like native Tnt1 elements, mini-Tnt1 s insert within or near genes and transpose at frequencies comparable with native Tnt1 elements; (2) mini-Tnt1 s can be engineered to carry heterologous genes (for example, marker genes) to facilitate identification of cells with transposition events; (3) after transformation, helper elements can be removed by backcrossing to turn off transposition; and (4) half of the transposed mini-Tnt1 elements integrate into the genome shortly after transformation, even before the parental mini-Tnt1 copies, thereby simplifying recovery and analysis of mini-Tnt1 integration sites. The latter observation was also made in *M. truncatula*, in which approximately 11% of transformants contain newly transposed Tnt1 elements but lack the transforming T-DNA [12].

We are particularly intrigued by the possibility of using mini-Tnt1 s to produce cDNA that can engage in homologous recombination with related chromosomal sequences. Gene modification through homologous recombination has been difficult to achieve in plants, although important advances have recently been made by creating targeted chromosome breaks with engineered nucleases. Although chromosome break stimulates recombinational repair, a donor DNA molecule must still be introduced into cells to serve as a repair template. The two most widely used methods for introducing DNA into plants are infection by *Agrobacterium *and high-velocity particle bombardment. In both methods, the input DNA remains in the plant cell for only a short period of time. Because homologous recombination is infrequent, transient delivery of DNA may not be ideal for efficient recombination. LTR retrotransposon cDNA can engage in homologous recombination in *S. cerevisiae *[27], and mini-Tnt1 cDNA should also be able to enter the genome by homologous recombination. We predict that sustained delivery of donor cDNAs by retrotransposon vectors will increase frequencies of homologous recombination.

## Conclusions

We have developed a retrotransposon vector system based on the tobacco Tnt1 element. Mini-Tnt1 transfer vectors can be mobilized in *trans *by GAG and POL produced by endogenous Tnt1 elements. This study opens the door for the use of mini-Tnt1 elements for transgene delivery and mutagenesis. Mini-Tnt1 elements also offer an easily manipulatable system for studying basic mechanisms of retrotransposition in plants.

## Methods

### DNA constructs

A full-length Tnt1 element was assembled from four different PCR products derived from amplification of *N. tabacum *cv. Xanthi genomic DNA. Sequence mismatches relative to the reference sequence (GenBank accession number X13777) were corrected by PCR-based mutagenesis. The full-length element was cloned into vector pDW860 (unpublished) between *Cla*I and A*pa*I sites to create plasmid pIP57. The mini-Tnt1 element (pIP62) was created by cloning the *Mlu*I-*Apa*I fragment (containing the 3' LTR and 324 bp of internal domain sequence) into pDW860 to generate pJR10. A three-fragment ligation was then performed using (1) a *Cla*I-*Xho*I fragment (containing the Tnt1 5' LTR and sequences extending into the first 315 bp of *GAG*), (2) a *Xho*I-*Mlu*I polylinker, and (3) pJR10 digested with *Cla*I and *Mlu*I. The 35S-mini-Tnt1 was created by first replacing the 233 nucleotides closest to the 5' end of the 5' LTR with the CaMV 35 S promoter by PCR-based mutagenesis. The *Cla*I-*Xba*I fragment containing the 35 S LTR was used to replace the corresponding fragment in pIP62 to yield pIP65. The DNA sequences of the mini-Tnt1 vectors are available in Additional file 1.

To provide a marker to follow transposition, an 85 bp artificial intron (5'-GTAAGTTTATCAGTTAAATATAATAAATAAAGAAGAAAACCAAAAAAATGGCTAACTAAAACGATGGTCTTATGATTTTATGCAG-3') was inserted into NPTII, and then the modified NPTII fragment was fused to the NOS promoter. The *Xho*I-*Nde*I fragment containing the NOS-NPTII construct was inserted into pIP62 and pIP65 to yield pYH175 and pJR17, respectively. pJR17 was digested with *Bgl*II and *pfl*M1, and the resulting fragment was treated with mung bean nuclease and re-ligated with T4 DNA ligase to yield the PBS deletion mutant pYH176. Similarly, pYH175 was digested with *Xba*I and *pfl*M1, treated with mung bean nuclease and re-ligated to create the PBS deletion mutant pYH178. PCR-based mutagenesis was used to delete the PPT. The *Nde*I-*Apa*I PCR fragment with the PPT deletion was used to replace the corresponding fragment in pYH176 to yield pYH229. The same strategy was applied to pYH178 to make pYH233, which has both the PBS and PPT deletions.

### Plant transformation and plant material

Transformed tobacco calli were obtained by culturing electroporated protoplasts isolated from leaf mesophyll cells of *N. tobaccum *cv. Xanthi., using the electroporation protocol described by Wright *et al*.[28]. The culture media contained kanamycin 50 mg/ml. Numbers of Kan^r ^calli were scored after 60 days. Genomic DNA was prepared from calli with a DNA extraction kit (Epicentre Biotechnologies, Madison, WI, USA).

### Inverse PCR

Two nested primer pairs were used to amplify the sequences flanking either the 5' or 3' LTRs of new mini-Tnt1 insertions. (for primer sequences, see Additional file 1). Genomic DNA (200 ng) was digested with *Hae*III in a 20 μl reaction volume for 12 hours at 37°C. After heat inactivation of the restriction enzyme (20 min at 80°C), the DNA was self-ligated at 15°C overnight in 100 μl of ligase buffer containing 40 U ligase. The ligation products (5 μl) were used in subsequent PCR reactions. The first reaction was carried out in a 50 μl volume of 1× Jumpstart Taq buffer, 0.75 μl of each primer (20 mM), 8 μl 2.5 mM dNTPs and 2.5 U Jumpstart Taq polymerase (Sigma Aldrich, St Louis, MO, USA). The product of the first PCR reaction was diluted 100-fold, and 1 μl of diluted product was used in the next round of PCR with the second set of PCR primers.

### Sequence analysis

Sequences flanking mini-Tnt1 s were used as queries in BLASTN, BLASTX or TBLASTX searches against the non-redundant (nr) and EST databases at the National Center for Biotechnology information (NCBI, http://www.ncbi.nlm.nih.gov) or against the tobacco gene index (NtGI, http://compbio.dfci.harvard.edu/tgi/cgi-bin/tgi/gimain.pl?gudb=tobacco). Flanking sequences that returned high scoring pairs with e-values < 1.00E-5 were considered as significant.

## Competing interests

The authors declare that they have no competing interests.

## Authors' contributions

YH obtained and interpreted all of the data for the mini-Tnt1 elements. She also made several of the constructs and prepared the first draft of the manuscript. JR made some of the initial mini-Tnt1 constructs and performed preliminary tests that demonstrated mini-Tnt1 function in tobacco and *Arabidopsis*. PI generated a full-length Tnt1 element and helped make mini-Tnt1 constructs. DV supervised the research, obtained funding for the work and helped to draft the manuscript. All authors read and approved the final manuscript.

## Supplementary Material

Additional file 1 **Supplemental Data and information**. Click here for file
