# Supplementary material for: Retrotransposon vectors for gene delivery in plants
Source: Mob DNA. 2010 Aug 2;1:19. doi: 10.1186/1759-8753-1-19 (PMC2923131; doi:10.1186/1759-8753-1-19)
Supplement: Additional file 1 — Supplemental Data and information. [file 1759-8753-1-19-S1.DOCX]

**Supplemental Data and information**

**Retrotransposon vectors for gene delivery in plants**

Yi Hou, Jyothi Rajagopal, Phillip A. Irwin and Daniel F. Voytas*

Department of Genetics, Cell Biology and Development and Center for Genome Engineering, University of Minnesota, Minneapolis, Minnesota USA 55455

Email: Voytas@umn.edu

**pIP62 Wild type mini-Tnt1 2034bp:**

**1-6: Unique ClaI cloning site for removal of the entire mini-Tnt1 cassette**

**7-616: Tnt1 5’ LTR**

**617- 697: non-coding leader sequence**

**698-1012: Translation start and Tnt1 GAG coding sequence**

**1013-1095: Multiple cloning sites**

**1096-1372: C-terminus of Tnt1 POL up to the stop codon**

**1373-1418: Tnt1 non-coding sequence between stop codon and 3’ LTR**

**1419-2028: Tnt1 3’ LTR**

**2029-2034: Unique ApaI Cloning site for removal of the entire mini-Tnt1 cassette**

**5’ ATCGATTGATGATGTCCATCTCATTGAAGAAGTA**

**TTAGGCATGTGCCTAATAAGAGTTTTCTTTGGTTTGGTAGCCAACCTTGT**

**TGACTTGGTTTGGTTGGTAGCCAACCTTGTTGAATCCTTGTTGGATTGGT**

**AGCCAACTTTGTTGAATTGTGAAAAATGTGTGTAAATTGTCAAATATTGT**

**AGGCTTTAGAGGGTGAAGCTTTGGCTATAAAAGGAGAGCTTCAACTCTCA**

**TTTCTTCACACCAACAAAGAGAGAAAGAAAGAGTGAGGTTTCACAGACAA**

**GGTATAAGAAAATAGTCTGTGAGGAAAATAGAGAGTGAGCGATATTGTAG**

**TGAGGTGGGAATATCAAAAGAGGGTTATTTCTTTTGAGTGTTGTAGTGGT**

**CTTTGGAGTATTTACCTCCGACCTACAAAGTGTAAAATTCCTTACTATAG**

**TGATATCAGTTGCTCCTCTCGGGGTCGTGGTTTTTTTTCCCTTATTCAGA**

**AGGGTTTTCCACGTAAAAATCTTGGTGTCATTGTTACTCTTTTATTCTTG**

**TTAATTACCGTATCTCGGTGCTACATTATTATTCCGCTTTATTACCGTGA**

**ATATTATTTTGGTAAGGGGTTTATTCCCAACAACTGGTATCAGAGCACAG**

**GTTCTGCTCGTTCACTGAAATACTATTCACTGTCGGTAGTACTATACTTG**

**GTGAAAAATAAAAATGTCTGGAGTAAAGTACGAGGTAGCAAAATTCAATG**

**GAGATAACGGTTTCTCAACATGGCAAAGAAGGATGAGAGATCTGCTCATC**

**CAACAAGGATTACACAAGGTTCTAGATGTTGATTCCAAAAAGCCTGATAC**

**CATGAAAGCTGAGGATTGGGCTGACTTGGATGAAAGAGCTGCTAGTGCAA**

**TCAGGTTGCACTTATCAGATGATGTGGTAAATAACATCATTGATGAAGAC**

**ACTGCACGTGGAATTTGGACAAGGTTGGAAAGCCTATACATGTCCAAAAC**

**GCTGACAAATAAATTGTACCTGAAGAAGCTCGAGCGTACGGCGCCGGATC**

**CGAATTCTCCCCGCGGAGGTACCTCCCCCGGGATGAGCTCTACTAGTAAA**

**CGCGTCATATGCAAGCGATTCCTTCAAGAGCTTGGATTGCATCAGAAGGA**

**GTATGTCGTCTATTGTGACAGTCAAAGTGCAATAGACCTTAGCAAGAACT**

**CTATGTACCATGCAAGGACCAAACACATTGATGTGAGATATCATTGGATT**

**CGAGAAATGGTAGATGATGAATCTCTAAAAGTCTTGAAGATTTCTACAAA**

**TGAGAATCCCGCAGATATGCTGACCAAGGTGGTACCAAGGAACAAGTTCG**

**AGCTATGCAAAGAACTTGTCGGCATGCATTCAAACTAGAAGACAGTGCTA**

**CCTCCTCTGGATGAATGAGACTGGAGGGGGAGATTGATGATGTCCATCTC**

**ATTGAAGAAGTATTAGGCATGTGCCTAATAAGAGTTTTCTTTGGTTTGGT**

**AGCCAACCTTGTTGACTTGGTTTGGTTGGTAGCCAACCTTGTTGAATCCT**

**TGTTGGATTGGTAGCCAACTTTGTTGAATTGTGAAAAATGTGTGTAAATT**

**GTCAAATATTGTAGGCTTTAGAGGGTGAAGCTTTGGCTATAAAAGGAGAG**

**CTTCAACTCTCATTTCTTCACACCAACAAAGAGAGAAAGAAAGAGTGAGG**

**TTTCACAGACAAGGTATAAGAAAATAGTCTGTGAGGAAAATAGAGAGTGA**

**GCGATATTGTAGTGAGGTGGGAATATCAAAAGAGGGTTATTTCTTTTGAG**

**TGTTGTAGTGGTCTTTGGAGTATTTACCTCCGACCTACAAAGTGTAAAAT**

**TCCTTACTATAGTGATATCAGTTGCTCCTCTCGGGGTCGTGGTTTTTTTT**

**CCCTTATTCAGAAGGGTTTTCCACGTAAAAATCTTGGTGTCATTGTTACT**

**CTTTTATTCTTGTTAATTACCGTATCTCGGTGCTACATTATTATTCCGCT**

**TTATTACCGTGAATATTATTTTGGTAAGGGGTTTATTCCCAACAGGGCCC3’**

**pIP65 35S mini-Tnt1 2681bp:**

**1-56: Linker sequence with unique ClaI and BsiWI sites for removal of the entire mini-Tnt1 cassette**

**57-886: 35S promoter region**

**887-1263: Tnt1 5’ LTR**

**1264-1344: non coding leader sequence**

**1345-1659: Translation start and Tnt1 GAG coding sequence**

**1660-1742: Multiple cloning sites**

**1743-2019: C-terminus of Tnt1 POL up to the stop codon**

**2020-2065: Tnt1 non-coding sequence between stop codon and 3’ LTR**

**2066-2675: Tnt1 3’ LTR**

**2676-2681: Unique ApaI Cloning site for removal of the entire mini-Tnt1 cassette**

**5’ ATCGATGGCGCCAGCTGCAGGAATTCGATATCAA**

**GCTTATCGATCGTACGGTCCCCAGATTTGCCTTTTCAATTTCAGAAAGAA**

**TGCTAACCCACAGATGGTTAGAGAGGCTTACGCAGCAGGTCTCATCAAGA**

**CGATCTACCCGAGCAATAATCTCCAGGAAATCAAATACCTTCCCAAGAAG**

**GTTAAAGATGCAGTCAAAAGATTCAGGACTAACTGCATCAAGAACACAGA**

**GAAAGATATATTTCTCAAGATCAGAAGTACTATTCCAGTATGGACGATTC**

**AAGGCTTGCTTCACAAACCAAGGCAAGTAATAGAGATTGGAGTCTCTAAA**

**AAGGTAGTTCCCACTGAATCAAAGGCCATGGAGTCAAAGATTCAAATAGA**

**GGACCTAACAGAACTCGCCGTAAAGACTGGCGAACAGTTCATACAGAGTC**

**TCTTACGACTCAATGACAAGAAGAAAATCTTCGTCAACATGGTGGAGCAC**

**GACACACTTGTCTACTCCAAAAATATCAAAGATACAGTCTCAGAAGACCA**

**AAGGGCAATTGAGACTTTTCAACAAAGGGTAATATCCGGAAACCTCCTCG**

**GATTCCATTGCCCAGCTATCTGTCACTTTATTGTGAAGATAGTGGAAAAG**

**GAAGGTGGCTCCTACAAATGCCATCATTGCGATAAAGGAAAGGCCATCGT**

**TGAAGATGCCTCTGCCGACAGTGGTCCCAAAGATGGACCCCCACCCACGA**

**GGAGCATCGTGGAAAAAGAAGACGTTCCAACCACGTCTTCAAAGCAAGTG**

**GATTGATGTGATATCTCCACTGACGTAAGGGATGACGCACAATCCCACTA**

**TCCTTCGCAAGACCCTTCCTCTATATAAGGAAGTTCATTTCATTTGGAGA**

**GATCACACCAACAAAGAGAGAAAGAAAGAGTGAGGTTTCACAGACAAGGT**

**ATAAGAAAATAGTCTGTGAGGAAAATAGAGAGTGAGCGATATTGTAGTGA**

**GGTGGGAATATCAAAAGAGGGTTATTTCTTTTGAGTGTTGTAGTGGTCTT**

**TGGAGTATTTACCTCCGACCTACAAAGTGTAAAATTCCTTACTATAGTGA**

# TATCAGTTGCTCCTCTCGGGGTCGTGGTTTTTTTTCCCTTATTCAGAAGG

**GTTTTCCACGTAAAAATCTTGGTGTCATTGTTACTCTTTTATTCTTGTTA**

## ATTACCGTATCTCGGTGCTACATTATTATTCCGCTTTATTACCGTGAATA

**TTATTTTGGTAAGGGGTTTATTCCCAACAACTGGTATCAGAGCACAGGTT**

**CTGCTCGTTCACTGAAATACTATTCACTGTCGGTAGTACTATACTTGGTG**

**AAAAATAAAAATGTCTGGAGTAAAGTACGAGGTAGCAAAATTCAATGGAG**

**ATAACGGTTTCTCAACATGGCAAAGAAGGATGAGAGATCTGCTCATCCAA**

**CAAGGATTACACAAGGTTCTAGATGTTGATTCCAAAAAGCCTGATACCAT**

**GAAAGCTGAGGATTGGGCTGACTTGGATGAAAGAGCTGCTAGTGCAATCA**

**GGTTGCACTTATCAGATGATGTGGTAAATAACATCATTGATGAAGACACT**

**GCACGTGGAATTTGGACAAGGTTGGAAAGCCTATACATGTCCAAAACGCT**

**GACAAATAAATTGTACCTGAAGAAGCTCGAGCGTACGGCGCCGGATCCGA**

**ATTCTCCCCGCGGAGGTACCTCCCCCGGGATGAGCTCTACTAGTAAACGC**

**GTCATATGCAAGCGATTCCTTCAAGAGCTTGGATTGCATCAGAAGGAGTA**

**TGTCGTCTATTGTGACAGTCAAAGTGCAATAGACCTTAGCAAGAACTCTA**

**TGTACCATGCAAGGACCAAACACATTGATGTGAGATATCATTGGATTCGA**

**GAAATGGTAGATGATGAATCTCTAAAAGTCTTGAAGATTTCTACAAATGA**

**GAATCCCGCAGATATGCTGACCAAGGTGGTACCAAGGAACAAGTTCGAGC**

**TATGCAAAGAACTTGTCGGCATGCATTCAAACTAGAAGACAGTGCTACCT**

**CCTCTGGATGAATGAGACTGGAGGGGGAGATTGATGATGTCCATCTCATT**

**GAAGAAGTATTAGGCATGTGCCTAATAAGAGTTTTCTTTGGTTTGGTAGC**

**CAACCTTGTTGACTTGGTTTGGTTGGTAGCCAACCTTGTTGAATCCTTGT**

**TGGATTGGTAGCCAACTTTGTTGAATTGTGAAAAATGTGTGTAAATTGTC**

**AAATATTGTAGGCTTTAGAGGGTGAAGCTTTGGCTATAAAAGGAGAGCTT**

**CAACTCTCATTTCTTCACACCAACAAAGAGAGAAAGAAAGAGTGAGGTTT**

**CACAGACAAGGTATAAGAAAATAGTCTGTGAGGAAAATAGAGAGTGAGCG**

**ATATTGTAGTGAGGTGGGAATATCAAAAGAGGGTTATTTCTTTTGAGTGT**

**TGTAGTGGTCTTTGGAGTATTTACCTCCGACCTACAAAGTGTAAAATTCC**

**TTACTATAGTGATATCAGTTGCTCCTCTCGGGGTCGTGGTTTTTTTTCCC**

**TTATTCAGAAGGGTTTTCCACGTAAAAATCTTGGTGTCATTGTTACTCTT**

**TTATTCTTGTTAATTACCGTATCTCGGTGCTACATTATTATTCCGCTTTA**

**TTACCGTGAATATTATTTTGGTAAGGGGTTTATTCCCAACAGGGCCC3’**

**35S Mini Tnt1 with NPTII gene**

**1-12: Linker sequence**

**12-842: 35S promoter**

**843-1219: Partial sequence of Tnt1 LTR; position 843 is the Tnt1 transcriptional start site**

**1220-1300: non-coding leader sequence**

**1301-1615: Translation start and Tnt1 Gag coding sequence**

**1616-1621: XhoI site (in lower case)**

**1622-2825: NOS promoter and the NPTII gene with the intron (lower case)**

**2826-2831: Nde1 site (lower case)**

**2832-3155: C-terminus of Tnt1 POL and non-coding sequence between stop codon and 3’ LTR**

**3156-3765: 3’ Tnt1 LTR**

**3766-3771: ApaI cloning site**

**CGTACGGTCCCCAGATTTGCCTTTTCAATTTCAGAAAGAATGCTAACCCA**

**CAGATGGTTAGAGAGGCTTACGCAGCAGGTCTCATCAAGACGATCTACCC**

**GAGCAATAATCTCCAGGAAATCAAATACCTTCCCAAGAAGGTTAAAGATG**

**CAGTCAAAAGATTCAGGACTAACTGCATCAAGAACACAGAGAAAGATATA**

**TTTCTCAAGATCAGAAGTACTATTCCAGTATGGACGATTCAAGGCTTGCT**

**TCACAAACCAAGGCAAGTAATAGAGATTGGAGTCTCTAAAAAGGTAGTTC**

**CCACTGAATCAAAGGCCATGGAGTCAAAGATTCAAATAGAGGACCTAACA**

**GAACTCGCCGTAAAGACTGGCGAACAGTTCATACAGAGTCTCTTACGACT**

**CAATGACAAGAAGAAAATCTTCGTCAACATGGTGGAGCACGACACACTTG**

**TCTACTCCAAAAATATCAAAGATACAGTCTCAGAAGACCAAAGGGCAATT**

# GAGACTTTTCAACAAAGGGTAATATCCGGAAACCTCCTCGGATTCCATTG

**CCCAGCTATCTGTCACTTTATTGTGAAGATAGTGGAAAAGGAAGGTGGCT**

**CCTACAAATGCCATCATTGCGATAAAGGAAAGGCCATCGTTGAAGATGCC**

**TCTGCCGACAGTGGTCCCAAAGATGGACCCCCACCCACGAGGAGCATCGT**

**GGAAAAAGAAGACGTTCCAACCACGTCTTCAAAGCAAGTGGATTGATGTG**

**ATATCTCCACTGACGTAAGGGATGACGCACAATCCCACTATCCTTCGCAA**

**GACCCTTCCTCTATATAAGGAAGTTCATTTCATTTGGAGAGATCACACCA**

**ACAAAGAGAGAAAGAAAGAGTGAGGTTTCACAGACAAGGTATAAGAAAAT**

**AGTCTGTGAGGAAAATAGAGAGTGAGCGATATTGTAGTGAGGTGGGAATA**

**TCAAAAGAGGGTTATTTCTTTTGAGTGTTGTAGTGGTCTTTGGAGTATTT**

**ACCTCCGACCTACAAAGTGTAAAATTCCTTACTATAGTGATATCAGTTGC**

**TCCTCTCGGGGTCGTGGTTTTTTTTCCCTTATTCAGAAGGGTTTTCCACG**

### TAAAAATCTTGGTGTCATTGTTACTCTTTTATTCTTGTTAATTACCGTAT

**CTCGGTGCTACATTATTATTCCGCTTTATTACCGTGAATATTATTTTGGT**

**AAGGGGTTTATTCCCAACAACTGGTATCAGAGCACAGGTTCTGCTCGTTC**

**ACTGAAATACTATTCACTGTCGGTAGTACTATACTTGGTGAAAAATAAAA**

**ATGTCTGGAGTAAAGTACGAGGTAGCAAAATTCAATGGAGATAACGGTTT**

**CTCAACATGGCAAAGAAGGATGAGAGATCTGCTCATCCAACAAGGATTAC**

**ACAAGGTTCTAGATGTTGATTCCAAAAAGCCTGATACCATGAAAGCTGAG**

**GATTGGGCTGACTTGGATGAAAGAGCTGCTAGTGCAATCAGGTTGCACTT**

**ATCAGATGATGTGGTAAATAACATCATTGATGAAGACACTGCACGTGGAA**

**TTTGGACAAGGTTGGAAAGCCTATACATGTCCAAAACGCTGACAAATAAA**

**TTGTACCTGAAGAAGctcgagGGTACCGGATCATGAGCGGAGAATTAAGG**

**GAGTCACGTTATGACCCCCGCCGATGACGCGGGACAAGCCGTTTTACGTT**

**TGGAACTGACAGAACCGCAACGTTGAAGGAGCCACTGAGCCGCGGGTTTC**

**TGGAGTTTAATGAGCTAAGCACATACGTCAGAAACCATTATTGCGCGTTC**

**AAAAGTCGCCTAAGGTCACTATCAGCTAGCAAATATTTCTTGTCAAAAAT**

**GCTCCACTGACGTTCCATAAATTCCCCTCGGTATCCAATTAGAGTCTCAT**

**ATTCACTCTCAATCCAGATCTGATCATGTGGATTGAACAAGATGGATTGC**

**ACGCAGGTTCTCCGGCCGCTTGGGTGGAGAGGCTATTCGGCTATGACTGG**

**GCACAACAGACAATCGGCTGCTCTGATGCCGCCGTGTTCCGGCTGTCAGC**

**GCAGGGGCGCCCGGTTCTTTTTGTCAAGACCGACCTGTCAGgtaagttta**

**tcagttaaatataataaataaagaagaaaaccaaaaaaatggctaactaa**

**aacgatggtcttatgattttatgcagGTGCCCTGAATGAACTGCAGGACG**

**AGGCAGCGCGGCTATCGTGGCTGGCCACGACGGGCGTTCCTTGCGCAGCT**

**GTGCTCGACGTTGTCACTGAAGCGGGAAGGGACTGGCTGCTATTGGGCGA**

**AGTGCCGGGGCAGGATCTCCTGTCATCTCACCTTGCTCCTGCCGAGAAAG**

**TATCCATCATGGCTGATGCAATGCGGCGGCTGCATACGCTTGATCCGGCT**

**ACCTGCCCATTCGACCACCAAGCGAAACATCGCATCGAGCGAGCACGTAC**

**TCGGATGGAAGCCGGTCTTGTCGATCAGGATGATCTGGACGAAGAGCATC**

**AGGGGCTCGCGCCAGCCGAACTGTTCGCCAGGCTCAAGGCGCGCATGCCC**

# GACGGCGAGGATCTCGTCGTGACCCATGGCGATGCCTGCTTGCCGAATAT

**CATGGTGGAAAATGGCCGCTTTTCTGGATTCATCGACTGTGGCCGGCTGG**

**GTGTGGCGGACCGCTATCAGGACATAGCGTTGGCTACCCGTGATATTGCT**

**GAAGAGCTTGGCGGCGAATGGGCTGACCGCTTCCTCGTGCTTTACGGTAT**

**CGCCGCTCCCGATTCGCAGCGCATCGCCTTCTATCGCCTTCTTGACGAGT**

**TCTTCTGAGCGGGACTCTGGGGTTCcatatgTCAAGCGATTCCTTCAAGA**

**GCTTGGATTGCATCAGAAGGAGTATGTCGTCTATTGTGACAGTCAAAGTG**

**CAATAGACCTTAGCAAGAACTCTATGTACCATGCAAGGACCAAACACATT**

**GATGTGAGATATCATTGGATTCGAGAAATGGTAGATGATGAATCTCTAAA**

**AGTCTTGAAGATTTCTACAAATGAGAATCCCGCAGATATGCTGACCAAGG**

**TGGTACCAAGGAACAAGTTCGAGCTATGCAAAGAACTTGTCGGCATGCAT**

**TCAAACTAGAAGACAGTGCTACCTCCTCTGGATGAATGAGACTGGAGGGG**

**GAGATTGATGATGTCCATCTCATTGAAGAAGTATTAGGCATGTGCCTAAT**

**AAGAGTTTTCTTTGGTTTGGTAGCCAACCTTGTTGACTTGGTTTGGTTGG**

**TAGCCAACCTTGTTGAATCCTTGTTGGATTGGTAGCCAACTTTGTTGAAT**

**TGTGAAAAATGTGTGTAAATTGTCAAATATTGTAGGCTTTAGAGGGTGAA**

**GCTTTGGCTATAAAAGGAGAGCTTCAACTCTCATTTCTTCACACCAACAA**

**AGAGAGAAAGAAAGAGTGAGGTTTCACAGACAAGGTATAAGAAAATAGTC**

**TGTGAGGAAAATAGAGAGTGAGCGATATTGTAGTGAGGTGGGAATATCAA**

**AAGAGGGTTATTTCTTTTGAGTGTTGTAGTGGTCTTTGGAGTATTTACCT**

**CCGACCTACAAAGTGTAAAATTCCTTACTATAGTGATATCAGTTGCTCCT**

**CTCGGGGTCGTGGTTTTTTTTCCCTTATTCAGAAGGGTTTTCCACGTAAA**

**AATCTTGGTGTCATTGTTACTCTTTTATTCTTGTTAATTACCGTATCTCG**

**GTGCTACATTATTATTCCGCTTTATTACCGTGAATATTATTTTGGTAAGG**

# GGTTTATTCCCAACAGGGCCC

**PCR primers:**

**Primer for testing intron loss:**

| Primer | Sequence |
| --- | --- |
| DVO4576 | 5' TCG GCT ATG ACT GGG CAC AA 3' |
| DVO4577 | 5' CGG CAG GAG CAA GGT GAG AT 3' |

**Primers for IPCR:**

| Primer | Sequence |
| --- | --- |
| DVO4707 | 5' GCC AAA GCT TCA CCC TCT AAA GCC TAC A 3' |
| DVO4782 | 5' GCT ACC AAC CAA ACC AAG TCA AC 3' |
| DVO4783 | 5' ATC TCG GTG CTA CAT TAT TAT TC 3' |
| DVO4874 | 5' CGT TCC ATA AAT TCC CCT CGG TAT CCA 3' |
| DVO4877 | 5' AGT TGC TCC TCT CGG GGT CGT 3' |
| DVO4878 | 5' CCC CAG AGT CCC GCT CAG AAG A 3' |
| DVO4880 | 5' CAC GGG TAG CCA ACG CTA TGT CCT 3' |
| DVO4944 | 5' GGT TGG CTA CCA ACC AAA CCA AGT CAA CAA 3' |

Additional results of PCR screens for intron loss in kanamycin resistant calli derived from various mini-Tnt1s. Lanes 1 & 11, DNA markers; lane 2, negative control, namely the parental mini-Tnt1; lane 3, positive control, namely a plasmid carrying NPTII without an intron; lane 4, control without template; lanes 7, 13 & 15, intron loss; lanes 5, 6, 10, 12, 16, 17, 18 & 19, intron retention; lanes 8, 9 14 & 20, both intron loss and retention.


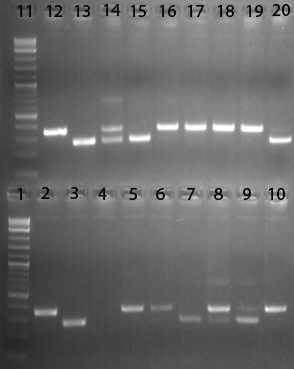


**DNA sequences of mini-Tnt1 Insertion sites (red sequences are the target site):**

**pJR17 (1) 7G**

>insertion site

GGCCAATCGATCCTAATTCAATTATACGGCTGGGTTNTAAACCAAAAAAGGGCATTTGAATGCAGAGCAAGTATTAGTAGCATAACAGAAAAGCCTTCCTNGTCATTAATATCTTTGCTCGCGGCAATTGTGACCTNTCGGGAGAATCGATTACTGCATCAAAGATGCAGTGCTAGTGCATTTGAGACTTNTTAATTGGCTAGTTGTAAATAGCCCCAGGGCTATGGAAAAAAGGATTATATCGGACCTAGACCGAAGTGTTGATGGTGATTTTTTAATCTCGCAGAATAGAATGNGAATAAGTCAATTTGTACTNTTTAACCCCAGCTTNGAATACAACNCTTGCTTTAGTTTCTGTTTGTGGNGACANAAATTNCNCCCTACAACTCATGAATTAAGAATTTTCACAAAAACAAGGTTTACTNGACNNGAATTCCGCATTAATGAAACTTTTCCCAGGAATTTTTCNCAAAATTTCCAACTAATACTAATATTATCAACTAATAAGAATATTTGATTATTAGACCATTGGGACAGTATGGTATCCAGCCAGCTAAATTGACAATCTTAGCTAAAAGACACTCCGGAGACTAGACTTGTAGACTGACTTCCAGTTGTAGTACACAACTCTTAACCAAAAAACACTCCAAAAACTGATTGGTGTACAATGAATTTGATATCATTAAGTAGATCCTCTGAGATCATTGCTTTGCACTAACAATTCTTGTTTTAGCTCAACAAGGAAAAAGTAAAAAACCAGCCTGTTATAGTTATGGGCAATCTTCTCTTTGCTCTTGCAGTTTTCATTCTGCTACATGACCATACTTCCTTTGCTACTGTTCCCAATATTAGCACCGATGAAGCTGCTCTTCTTGCATTGAAATCTCACATTTCTTTTGGTCCTAATAATATCTTAGCAAGAAACTGGTCTTCTTCAGGCC

**pJR17 (2) 9A**

>insertion site

GGCCCAAATAAGCTATGGGGCTTACTAACGGGACTGAAATTTTCAAACTTGCATGATGAGTGGGAAACTACAGAAAAAATAAGGGCAACTAAAAGTTTAAAAGCATAGTTTAAAGGCAATTAATGGACNNTACGAAACATAAATGTGTTTAGAGATTTATTTTAACTTACCTAGATGTNTAGAGATTCACAAGAAATAAATATGTTAACGTGTCAAAATTGACAAGCNCCTCNCTAATTTCATTTTTTGGGAATTGCATTCACCANTTCCTTCTGTTCTCAACTATCCAATTTCGCTATATTGTTCCGACNTTAATTTAGTGGTGGAGACATCAGAATTTCAAATTTCATTTAGTGGTCACTATTAATGNNNNTGCCTCCCCCCCCCCCCCCCCNGTNANNCGGGNAAAAAAAAAAGAATTTTTTTTTTTTTTTTTTTGGNAAAAAAAAAAAAGGGGGGGNNGGATTTCTTCTAAAAAGGGGAAAAGGGGGGTGTNNNTTTTCCCCCCCCCCCAAATNTAAANTNTTCCTNATAATTNTGGGAATNTTGANGCNNAAAAA

**pJR17 (2) 9B**

>insertion site

GCGCCCCCAATTTTTTCCCCCCAAGTGTCCAATCAACGGCAATATTTGTTACTTGATAATTGATGGTGGTAGGGAAGAGAACATCATTGGAAAGAGTATTATTGAGAAACTACAGTTACCAGGGGAGAAACATTTGGATGTATCAAAACCGTAGGAAATTTCCAACTAACAAAAAGATGCAGAGTTTTTTTTTCAATTGGAAAGTACAAAGATGAAGTGTACTGCAACGTGGTAGACATGGANTCATCCCATCGGTTATTTCATAGGCTGTAGCAGTTTGACAAGGATGCTCGCCATTTTGGATGAAATAACATATATAAACTAGAAGTAAATGGNNNNAGGGTGAACCTAGTTCCAATGAATAAAATCAGTAAGCCCAAAGCTCGGAAAGTGGAGGCAAGTCCTTTGTGACACTTAGAGAAGGAGTTTGAAGTTGCTTGTAAGGACACTCAAGTAGTTCATTGCCTTGTGGTAAGAAAACATCCTAGTTACAGCAAAGGATCTACCAGAGAAATCAATTACCGATGATTTGAGGCTATTGTTAGAGGAGTTCAAGGATGTTATGCCCAAAGAGCTGCCTGCAGAGCTGCCACCTATACGAGATATTCAACACCACATTGATCCCATTCCTGGGGCAAGTTTACCAAACCTACCGCACTATTGAATGAGTCCTAATAAGAATGAGATTTTAAGCATCCAAGTTGAAGAGTTATTAAATAAAGGTCATATACCAGAGAGCATGAGCCCTTGTGCAGTACCAGCCTTACTCTCACCAAAGAAAGACAGAAGTTTGGAGAATGTGTGTAGACAGTAGGAAAACTAACAAGATCACTATGGGATATAAATTTCCAATTCCTTGATTTGAAGACATGCTGGACCAGTTAGATGGTTCAATATTATTTTCAAAGATTGATCTGCGC

**pYH175 (1) 2E**

>insertion site

GGCCAACAAAAGAAGAATCCCAGCAATTCAAGAACAAGAGAAATAGCAACCAACTAGAAATCCCATCAAAATTCGAGCAAAATGATATAGAATGAGAGAGGTTGAGGGAATGTGCCAGCATCCTCTTAAAATTAGGAAATTGGCATCTTCTGAAACAGAAAAATGGATAGCTATCCAGTTTTTGGATATTTCCTCTCACTCTCAACTCTTTCTTTAACAGATTGGGACACTTGTCCCTGTTTCTAGAAGCTTCTTTTATTCCCAGACCCTTTTAAATTTTTTAACTTCCAATTCTTACCCCAACATTTAAATTTATTCCACATTTTAATGTATTTCCAATTGAACAAAACAGATTCCTCCATTTTTGTCATTTCTTTTAGACCGCTGAATTTCAGTAATTACAAACAACTATCACAACAGAAAAAAACTCAGATAAATCAAAAATCACAGCACAACAAGCAATATTCAAACTTTAGCCTATTTAGGTGATTAAACGAATTCAAACCTACAATTAGCTAATCAAGATTACTTTGACCAAATTGAAACTGCGTACTATGCATCTAAAATTTTGGCC

**pYH175 (1) 2G**

>insertion site

GGCCCAATATTAATTTGTGTGACATCCTTGTAGGTATCCATATTGGGGTGGGCTAGATTATTCATAACAATACCCTCCTCCTCAATAAGAACCTTGTACTCAAGGTTCGAGTACATCAATACTAATGAGGAAAGACAATTGATATATGTTTGGGTTTCACACGGTAAAGAACATTTTGAATTATCCCGCAAAAGAAATCCCAAGATTTTCATCAGGACTTCTACAACCACCAAAAGAATTGATGCAATCCTTCAGGAGATATCAACTAAATAAAAAATAGGACTCACATAATATGCAGTAGCAGTAATCCATCCAAACTGATGAAATACACTTAAGACCACTTCCACTACAAGATAACGTGCATATTTTCTTTGAAATAATTCAGGCATCTTAGCCATTGAGTGTATTTGAAGGACTTCTCTGTATTTTAAAATTGCAGCAACTTCTATGGATATGTTGGAATTCTTTCCCCTAGGAGACGTTGGTATGGAAAATAGGTGGAGAATCTGTATTATAAATGTGTCATCCTTGTCTTTGTGGTCACTCTTTTGAAGGAGAGATAGCAATACCTGTTATGAGCACCTAATTTTTGACCATACTTGGGATTTATCGGCC

**pYH175 (2) 12H**

>insertion site

TGCATGAAGTCTGTTACGGAGAAGGATTTTGAGTGGTTGTCAAAGAATCCTAAAATTCTTGAAGCTAGTGTGATTATATGTCGAGTTATTGATGACACAGCCACATATGAGGTATGATCTGAGTTGTATCTCAGAAACTTATATAATTAAATGTGATTTGGACAAATGAAGTATTGCGACAACATTTGCGGCAATGTAAAAGCTAATGCCTAATCTATCTACCTTTTCAGGTTGAGAAAAGTAGGGGACAAATTGCAACAGGAATTGAGTGCTGCATGAGAGATTATGGCATATCAACAAACGAGGCAATGACTAAATTTCAAGAAATGGCTGAGGCAGCATGGAAGGACCTTAATGAAGGACTTCTTAGGCC

**The sequence of the reconstituted 35S-mini-Tnt1 5’LTR and 3’LTR after transposition (evidence for template switching between mini-Tnt1 and endogenous Tnt1 mRNA):**

**5’LTR**

NNNNNNNNNNNNNAATTTCNACTAATACTAATATTATCAACTAATAAGAATATTTGATTATTAGACCATTGGGACAGTATGGTATCTGATGATGTCCATCTCATTGAAGAAGTATTAGGCATGTGCCTAATAAGAGTTTTCTTTGGTTTGGTAGCCAACCTTGTTGACTTGGTTTGGTTGGTAGCCAACCTTGTTGAATTAGTTTGGTTTGGTAGCCAACCTTGTTGAATCCTTGTTGGATTGGTAGCCAACTTTGTTGAATTGTGAAAAATGTGTGTAAATTGTCAAATATTGTAGGCTTTAGAGGGTGAAGCTTTGGCTATAAAAGGAGAGCTTCAACTCTCATTTCTTCACACCAACAAAGAGAGAAAGAAAGAGTGAGGTTTCACAGACAAGGTATAAGAAAATAGTCTGTGAGGAAAATAGAGAGTGAGCGATATTGTAGTGAGGTGGGAATATCAAAAGAGGGTTATTTCTTTTGAGTGTTGTAGTGGTCTTTGGAGTATTTACCTCCGACCTACAAAGTGTAAAATTCCTTACTATAGTGATATCAGTTGCTCCTCTCGGGGTCGTGGTTTTTTTCCCCTTATTCAGAAGGGTTTTCCACGTAAAAATCTTGGTGTCATTGTTACTCTTTTATTCTTGTTAATTACCGTATCTCGGTGCTACATTATTATTCCGCTTTATTACCGTGAATATTATTTTGGTAAGGGGTTTATTCCCAACAACTGGTATCAAAGCACAGGTTCTGCTCGTTCACTGGCCGGACTGGA

**3’LTR**

AANNNNNNNTCNNTTCNTTGTACACCAATCAGTTTTTGGAGTGTTTTTTGGTTAAGAGTTGTGTACTACAACTGGAAGTCAGTCTACAAGTCTAGTCTCCGGAGTGTCTTTTAGCTAAGATTGTCAATTTAGCTGGCTGGATACTGTTGGGAATAAACCCCTTACCAAAATAATATTCACGGTAATAAAGCGGAATAATAATGTAGCACCGAGATACGGTAATTAACAAGAATAAAAGAGTAACAATGACACCAAGATTTTTACGTGGAAAACCCTTCTGAATAAGGGGAAAAAAACCACGACCCCGAGAGGAGCAACTGATATCACTATAGTAAGGAATTTTACACTTTGTAGGTCGGAGGTAAATACTCCAAAGACCACTACAACACTCAAAAGAAATAACCCTCTTTTGATATTCCCACCTCACTACAATATCGCTCACTCTCTATTTTCCTCACAGACTATTTTCTTATACCTTGTCTGTGAAACCTCACTCTTTCTTTCTCTCTTTGTTGGTGTGAAGAAATGAGAGTTGAAGCTCTCCTTTTATAGCCAAAGCTTCACCCTCTAAAGCCTACAATATTTGACAATTTACACACATTTTTCACAATTCAACAAAGTTGGCTACCAATCCAACAAGGATTCAACAAGGTTGGCTACCAAACCAAACTAATTCAACAAGGTTGGCTACCAACCAAACCAAGTCAACAAGGTTGGCTACCAAACCAAAGAAAACTCTTATTAGGCACATGCCTAATACTTCTTCAATGAGATGGACATCATCAATCTCCCCCTCCAGTCTCATTCATCTAGAGGAGGTAGCACTGTCTTCTAGTTTGAGTGCATGCCGANNNTTCTTTGCATAGCTCGAACTTGTTCCTTGGTACCACCTTGGNCAGCATATCTGCGGGATTCTCACTTGTAGAAATCTTCAAGACTTTTANAGATNCATCATCTACCATTTCTCGAATCNNNTGATNTCTCACATCAANGTGTTNGGNCNTGNATGNNCNTAGAGTNNNTGNNNNNNNNTGNACTTTGACTGTCANANAGANNNANNNCTNNNNNGATGCATNNNNTNTGNNNNNCNNTGNNNANGANNNNNNNCCN

**cDNA is synthesized when the mini-Tnt1 two component system is introduced into *Arabidopsis***

We introduced into Arabidopsis protoplasts both a 35S-mini-Tnt1 and a helper element in which Tnt1 *GAG-POL* was driven by the 35S promoter. Kanamycin resistant calli were selected and allowed to grow for 3 months. Two independent experiments produced 296 calli. The PCR-based assay was used to screen for intron loss, and 9 of 296 calli showed evidence of spliced cDNA (See examples in the figure below).


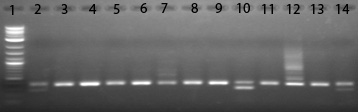


**Legend.** Data for mini-Tnt1 cDNA synthesis in *Arabidopsis thaliana.* PCR results for intron loss from mini-Tnt1. Lane 1 is a DNA marker; lanes 2, 10 & 14 are calli with two types of mini-Tnt1 elements, namely those with and without introns; lanes 3, 4, 5, 6, 7, 8, 9, 11, 12, & 13 are calli with mini-Tnt1 elements that show no evidence of intron loss.

**Methods**

*Arabidopsis* calli were obtained by culturing PEG-transformed protoplasts as described by Wenck and Marton, 1995. The *Arabidopsis thaliana* ecotype Columbia was grown under short-day conditions at 24C in germination media. At 4 to 6 days after germination, the seedlings were collected and treated with cellulase (0.25% W/V) and macerozyme (0.05% W/V) in K3 media (Nagy and Maliga, 1976) for 24 hours. Protoplasts were recovered by floatation in K3 media and washed once. PEG transformation of *Arabidopsis thaliana* was described in (Yoo et al., 2007). After transformation, the conditions for protoplast culture are the same as described in Wenck and Marton, 1995. Selection of protoplasts is described as in (Damm et al., 1989). Numbers of Kan^r^ calluses were scored after 90 days.

**Refences**

Damm, B., Schmidt, R., and Willmitzer, L. (1989). Efficient transformation of Arabidopsis thaliana using direct gene transfer to protoplasts. Mol. Gen. Genet. *217*, 6-12.

Nagy, J.I. and Maliga, P. (1976) Callus induction and plant regeneration from mesophyll protoplasts of Nicotiana sylvestris. Z. Pflanzenphysiol. *78*, 453–455.

Wenck, A.R., and Marton, L. (1995). Large-scale protoplast isolation and regeneration of Arabidopsis thaliana. Biotechniques *18*, 640-643.

Yoo, S.D., Cho, Y.H., and Sheen, J. (2007). Arabidopsis mesophyll protoplasts: a versatile cell system for transient gene expression analysis. Nat Protoc *2*, 1565-1572.
